# “*What can we do to actually reach all these animals*?” Evaluating approaches to improving working equid welfare

**DOI:** 10.1371/journal.pone.0273972

**Published:** 2022-09-09

**Authors:** Emily Haddy, Julia Brown, Faith Burden, Zoe Raw, Juliane Kaminski, Leanne Proops

**Affiliations:** 1 Centre for Comparative and Evolutionary Psychology, Department of Psychology, University of Portsmouth, Portsmouth, United Kingdom; 2 School of the Environment, Geography & Geosciences, University of Portsmouth, Portsmouth, United Kingdom; 3 The Donkey Sanctuary, Sidmouth, Devon, United Kingdom; Massey University, NEW ZEALAND

## Abstract

NGOs utilise a range of programming to bring about desired changes. This study examines the advantages and limitations of the range of approaches used by one particular NGO sector: working equid welfare organisations, from the perspective of NGO staff. NGO staff hold a wealth of knowledge regarding the design and implementation of welfare improvement initiatives; however this knowledge is rarely systematically documented. Through 32 semi-structured interviews the experiences of staff across multiple organisations were explored. The nine most commonly utilised approaches within equid focussed NGO programming were discussed with participants. Many themes resulting from the interviews centred around the strengths and disadvantages of these individual approaches, implemented both historically and presently by different NGOs. The influences of the context in which initiatives took place and barriers to welfare improvement that existed across approaches were also examined. Striking a balance came through as a strong theme at many levels. Balancing top-down versus bottom-up approaches was a frequently voiced concern, as was balancing the utility of certain approaches against associated factors warranting caution. Combinations of approaches that were felt to be complementary provided a balance that drew on the strengths and mitigated for the weaknesses of different approaches. The need to tailor approaches to individual contexts was also raised and is especially relevant to INGOs working across multiple countries, cultures and political structures. The study provides an informed insight into potential factors to consider when designing future welfare initiatives. The collaborative pooling of experience across different NGOs could help make welfare initiatives more effective and provide a framework for NGOs in other fields to learn from each other’s collective knowledge.

## Introduction

The approaches utilised by non-governmental organisations (NGOS) to bring about change vary based on factors such as the organisation’s size, mission and structure [1]. Organisations can be direct implementers (such as delivering aid in disaster affected areas or implementing humanitarian activities), purely advocacy-based (influencing policy and governmental decision making) or a hybrid of both [2]. Larger international NGOs likely employ multiple types of approaches whilst smaller specialist NGOs may focus solely on delivering one type of dedicated programming. Despite the variety of different subject areas that NGOs encompass, parallels can be drawn between the approaches used and programming decisions made across sectors. This study focuses on animal welfare NGOs, specifically those working to improve global welfare standards of working equids (donkeys, mules and horses). The diverse range of approaches used by these organisations to try and achieve welfare improvement has raised questions regarding the efficacy of these different models in achieving welfare change [3,4]. However there is little research evidence available to determine whether the effects of welfare initiatives endure in the long-term after programming ends [5]. Implementing an inappropriate type of initiative can lead to not only a lack of improvement in equid welfare but the loss of NGO resources and time, unmet expectations of target communities and potential disengagement with future welfare efforts [6]. Taking into consideration the existing debates regarding programming approaches, as well as the lack of published research evaluating the success of different types of welfare improvement initiatives [5–8]; the selection of an appropriate approach to implement can be difficult for practitioners. NGO staff hold a wealth of knowledge regarding the design and implementation of welfare improvement initiatives, including the contextual factors and practical barriers that impact success. However, this knowledge is rarely systematically documented. This study examines the advantages and limitations of the range of approaches used by one particular NGO sector: working equid welfare organisations, from the perspective of NGO staff across a range of positions. To the authors’ knowledge, this is the first study in the sector to explore the experiences of staff across multiple organisations, it is hoped that the collective experiences documented can aid all organisations when considering the design of future welfare initiatives.

Working equids are vital both economically and socially to millions of people worldwide. Contrary to the belief that with the increase of mechanisation working animals are declining in importance, recent figures show a rise in the number of working donkeys used worldwide, and a particular increase across sub-Saharan Africa [9]. Equids fulfil diverse roles, they offer a form of both traction and transport and are utilised across multiple types of industry [4]. They are of particular importance to marginalised sections of society and those in extreme poverty, sometimes representing a household’s only form of income [10]. Yet working equids commonly suffer from a high prevalence of welfare problems [11] and are largely absent from animal health and agricultural policy [10]. As a consequence, a range of organisations run programmes designed to improve the welfare of working equids and support the livelihoods of people relying on them. Originally, the provision of free or subsidised veterinary care was the most commonly utilised approach, however it is becoming increasingly acknowledged that the influences determining working equid welfare are complex and interlinked, with studies suggesting that socioeconomic and environmental factors both play important roles [11–13]. As a result, many NGOs are broadening the scope of their programming to target human factors and more actively engage with the people responsible for determining animal welfare. Approaches include introducing knowledge and skills to communities and helping to develop local infrastructure in order to support sustainable welfare improvements [14]. Increasing owner knowledge about animal welfare and sentience through targeted education programmes is also an avenue utilised for changing human attitudes towards animals [15]. In working with communities, the implementation of an initiative aiming to improve equid welfare relies entirely on community engagement and participation, therefore the inclusion of local perspectives and the addressing of socio-behavioural factors is crucial to ensuring initiative success [16]. Many NGOs are now using community focussed participatory exercise approaches to prioritise community views on welfare issues and use these perspectives to guide the direction of future programming [17]. At national and international levels, organisations are also engaging with the people responsible for creating animal welfare legislation and policy to create top-down changes that promote positive welfare.

## Methods

Semi-structured, online interviews were carried out with employees of animal welfare organisations, experienced in the development and implementation of equid welfare improvement initiatives. Interviews ran from February to July 2021 and a total of 32 individuals participated from 8 animal welfare organisations of varying sizes (INGO to national), participants were based in 13 countries. A representative range of opinions was sought within organisations (from office-based roles in headquarters to field staff working within equid-owning communities) and participants held positions including Directorial roles, Regional Managers and Co-ordinators, Veterinarians, Researchers and Welfare Officers. All individuals were involved with welfare approaches in some capacity, from those responsible for choosing the direction of programming through to those facilitating initiatives at ground level.

Researchers initially contacted two known staff members from two international working equid NGOS. These key contacts identified potential participants within their organisation and invitations to participate were sent to these individuals by the research team. The key contacts also identified key contacts in other relevant national and international NGOs who then facilitated participant recruitment within their organisation. Through this form of snowball sampling a volunteer sample of participants was recruited, all people who expressed their willingness to participate were interviewed. Interviews were pre-arranged, written consent was obtained and subsequently interviews took place online using Zoom video conferencing software. For subsequent transcription, all interviews were recorded and the average interview length was 55 minutes (30-108 minute range). Participants received a document detailing the interview themes before interviews were conducted (S1 File.) and a document listing the 9 most common approaches utilised by NGOs to improve working equid welfare (S2 File). The list provided was designed to be as exhaustive as possible and was informed by current NGO programming at the time; the option was also given for participants to discuss any non-listed approaches that they had encountered. Participants were asked about the approaches most often employed by their organisation (including any not listed) and whether they had noticed any changes in programming direction over time. They were also asked about their own past experiences carrying out welfare initiatives, particularly regarding instances which had been notably successful or unsuccessful, potential influencing factors and the context in which the initiative had taken place. A thematic analysis was conducted in order to explore similarities or differences in themes emerging from different approaches, organisations and staff positions. Themes were generated based on discussions of each approach separately. The 9 approaches in the list discussed during interviews were considered and reclassified during thematic analysis according to the manner in which they were most often grouped or referred to by participants. Although related, skills initiatives and education were frequently discussed by participants as separate programmes and with many points raised concerning each, it was decided to split these topics and present them separately. Conversely the topic ‘entertainment with a welfare message’ was mentioned most frequently in reference to education. As such, content relating to this topic is presented incorporated into the education section. Two non-listed approaches were discussed by participants, non-equid livelihoods-based programmes and disease surveillance programmes, discussion of these approaches has been integrated into the umbrella topic under which they fit. Themes related to common challenges which occurred across approaches were also noted and analysed. The University of Portsmouth’s Ethics Committee for the Faculty of Science and Health reviewed and approved the study (reference SHFEC 2020 – 087).

## Results and discussion

Through the process of thematic analysis major themes emerged from the data. Many of these themes centred around the strengths and weaknesses of the individual approaches implemented both historically and presently by different NGOs. The influences of the context in which initiatives took place and barriers to welfare improvement that exist across approaches were also examined. Each of these areas will be explored and prominent subthemes highlighted.

### Programmatic approaches to improving welfare

The nine most commonly utilised approaches within equid focussed NGO programming were explicitly discussed with participants. Approaches were considered in turn and the associated advantages, limitations and potential consequences of each were considered.

#### Advocacy

It was apparent that there has been a relatively recent shift placing advocacy as a strong focus across virtually all organisations. This is not unique to the working equid sector; the growth of advocacy across both generalist and specialist UK based NGOs has been documented [18]. A larger focus on advocacy was perceived as having both benefits and disadvantages. Some participants felt that advocating for policy change was the best way to enhance the lives of large numbers of animals, many more than could be reached through more practical, on the ground initiatives. *“I actually think targeting governments*, *and getting them to implement the [WOAH] chapters and getting them to enforce good welfare is probably your best way of making a huge difference*, *and having a huge reach*.*”* P14. It was recognised that advocacy can help change systems in a strategic way that aims to address root causes of poor welfare, rather than addressing the symptoms of individual animals. *“going for the right level would get better spread*, *better results*, *I think*, *in the end but it’s more of a long-term plan…firefighting is never going to get you a result at the end of the day*. *It’s not a strategic way of thinking*.*”* P14.

Recent advocacy efforts have seen equid welfare NGOs working together to form groups such as the International Coalition for Working Equids to implement the World Organisation for Animal Health (WOAH) Welfare of Working Equids Terrestrial Code Chapter 7.12 [19]. Other groups such as the Equid Power Network [20] have been involved in promoting educational events for the public and professionals across disciplines. It was acknowledged that for certain large-scale international problems (such as the trade in donkey skins [21]), correspondingly large-scale advocacy was likely to be the only effective solution. However, it was felt by some participants that the current prominence of advocacy on NGO agendas came at the expense of other approaches. *“I think*, *particularly nowadays*, *every charity organisation is actually advocating advocacy… It seems as if the practical work on the ground has been neglected”* P11. It was also expressed that advocacy could be essentially risky in terms of its rewards compared to the effort put in to achieve changes. Dependent upon the political climate, policy changes can be difficult to achieve and the process was described as being both lengthy and often frustrating for NGO staff. *“the least successful approach…(is) the engagement with the policymakers because these policymakers they are bureaucrats and the government systems are very slow”* P28. A second highlighted issue was that even if an advocacy strategy was successful in creating a policy change, this was no guarantee that the effect of the change would be felt by animals and communities. *“So that [law] existed when I first started working with the project and I don’t think I ever saw it enforced and I was there for 2 and a half years*. *So like although it was written down it didn’t make any difference on the ground as such*. *That’s just one of those things isn’t it*?*”* P2. A gap due to lack of enforcement of welfare legislation was often discussed as an obstacle to achieving welfare change. *“There is some policy on document*, *this policy has not been implemented*, *you know*, *these are really challenges for us*.*”* P25. A number of reasons were suggested for failure to enforce regulations including a lack of staff capacity, lack of resources at the disposal of the enforcing body and a lack of education of enforcers. *“policeman for example*. *Suddenly he is told that if he sees someone beating a donkey or carrying more than 300 kilos weight that man is… breaking the law*. *So he sees somebody carrying 200 kilos in the cart with 2 flat tyres*, *a bad harness and you know dogs biting the donkeys and everything else*. *And he’s not broken the rules so he lets him go*, *that’s the first problem*. *That the people that are enforcing the rules don’t understand what they are enforcing…without that knowledge they can’t make a judgement*.*”* P4. These factors are often systematic within enforcement entities (most commonly police or government enforcement bodies) which presents another challenge for NGOs who can have little control over how the policy is eventually enforced. This led to discussions that some political infrastructures are less likely to successfully support effective policy changes. *“lobbying and influencing future policy regarding equids can have greater and lasting impacts on welfare…mainly when the country is … resourceful enough to enforce it*.*”* P29.

Although acknowledging that it was resource intensive, some participants described an ideal balance needed of top down (advocacy based) and bottom-up (practical field based) programming. They felt that working from both directions was the most effective in terms of advocacy enabling higher up changes that supported an environment where good welfare could be created and sustained through on the ground programming. *“in an ideal world*, *you need to be doing advocacy*, *potentially*, *on a regional and international level*. *And then on all the subsequent levels down*, *right to the community*. *But you also need to be doing that community level work*, *and all the way up and having the combination of the two*.*”* P27. Continuing work on the ground alongside advocating enables immediate animal welfare needs to be addressed whilst informing advocacy efforts through having a thorough understanding of community level needs. *“I think it’s great that there’s a lot of organisations*, *I think*, *now moving to this more advocacy work*, *but I think*, *unless you know*, *the situation on the ground*, *I think it’s very difficult to advocate for those people and those horses*, *because you don’t know the context*.*”* P13.

#### Research

Organisations differed in the level of research that they carried out. Some considered research a priority and employed dedicated research teams to support their projects. *“I would focus on truly understanding the situation before you try to intervene”* P5. The investment of NGOs in research can be seen across sectors including animal welfare, international development and the environment, where research outputs are used to guide programming development [22–24]. The need for a thorough understanding of the community context, priorities and issues were underscored when planning a programme, especially if entering a new community. *“I really like how we got some decent research at the beginning of our project… we had an understanding of those places and that really helped to then design a better project from the word go*.*”* P2. Research was described as important both in informing the direction of programming by gathering contextual information about target communities and later ensuring that the approaches utilised were creating the desired changes. Although participants acknowledged that research could be a useful tool, some voiced feasibility concerns over the level of time and resources that research requires. *“I think it can be*, *it can be quite time consuming*. *And it can be quite expensive*, *as we all know*, *with research*. *But you know that investment is really worthwhile for that long term and sort of wider impact*.*”* P13. Size of organisation was also a factor that affected research engagement, with small national NGOs having limited ability to carry out their own research projects. *“Yeah*, *we’re not really big research people*. *I think it’s more capacity and time”* P23.

Where research was being used as a tool to identify prevalent welfare problems in an area, some participants expressed frustration. *“The issues for most of the working equids are pretty simple*, *so they are overworked*, *underfed and beaten*. *It doesn’t take a lot of research to see that*.*”* P4. This mainly centred on the time taken to conduct research on a subject that participants either felt was obvious to identify or could feasibly start to be addressed before research results were formalised. “*you know from the people or from what you see on the ground from the animals*, *what exactly the problem is*. *I think you have to start while … you are prioritising*, *while you are actually identifying the problems”* P11. Another issue identified by participants was not to do with the research process itself but the way in which research outputs were able to be used. *“actually I think that what is missing in research is a link between the university or the academic setting and the farm setting*, *the real life”* P1. Some felt that despite research being carried out, the findings were not routinely put into practice. *“I think the charity organisations they do one thing very nicely but that stops there when the research towards papers has been published*, *it goes there on the shelf*.*”* P11.

Planning for utilisation of research was described as important and some organisations only carried out research that they felt would be of direct practical value within their projects. Many participants described gaps where they felt further research was needed and across organisations three common topics were identified. Firstly, there was a call for research that provided further evidence on the socioeconomic contribution of equids to livelihoods. This was particularly mentioned as important for advocating for equids at a policy level. *“the socioeconomic value of the donkey and the donkey-owning people*, *what is the contribution of these people to the GDP of the country because it goes unnoticed*. *Of course imagine if the brick stops*? *Imagine all the houses*, *real-estate would collapse but people don’t know the bricks are made on the donkey back”* P8. Within the topic of livelihoods, it was also felt that evidence directly linking improvements in human livelihoods to animal welfare was needed. *“we have been promoting the livelihood of the communities*, *but we don’t have any evidence*, *scientific evidence to support that whether the increase in the livelihood improves the animal welfare or vice versa”* P28. Secondly, further information was required on infectious diseases of equids, a topic that has major welfare implications but was not felt to receive enough research attention. *“so many nasty diseases actually really hampering the health and welfare of equids in general in most developing countries”* P11. Within the topic of infectious diseases, zoonoses were included, an area likely to receive increased research attention in the wake of the COVID-19 pandemic. The final topic that was raised by multiple participants was the need for information or guidelines for the amount of weight animals should be carrying or pulling. *“how much weight can a donkey carry*, *that’s a question that comes up a lot*.*”* P9.

#### Veterinary clinics

The provision of free (or subsidised) veterinary care was the approach that generated the largest amount of discussion. *“I think it is one of those things that divides opinions the most I’m going to say running free vet clinics*.*”* P2. Whilst historically the most commonly used approach, a shift away from the provision of free veterinary clinics has been seen in recent years across many NGOs. A spectrum of opinions was expressed with large differences seen between the philosophies of individual organisations. The main concern regarding the provision of free veterinary clinics was that of sustainability. Criticisms both relating to the amount of resources necessary to run clinics and questions over the creation of lasting welfare change were expressed, mirroring debates in the literature [8,25]. *“[our organisation] cannot make sustainable scale level impact by veterinary service provision directly*. *There are too many donkeys and not enough vets and we wouldn’t be able to afford all the vets even if there were enough*.*”* P7. Creation of dependency on the service was a barrier to independent long-term welfare changes when NGO services are likely to, at some point, be withdrawn, enabling organisations to move to another location [7]. *“I think that’s always the difficulty when you go in*, *provide that free stuff*, *or subsidises*, *it’s much then harder to not provide it because it becomes an expectation*, *and I think we have to be aware of that”* P13. It was also expressed that free clinics, as an intensive approach, reached a limited number of animals in comparison to those able to be reached by other types of initiative. *“for years and years we may have worked in an area where the 200 donkeys in this immediate area are greatly benefitting from our weekly clinic but the other 2 million donkeys in this country never see us so what can we do to actually reach all these animals*?*”* P10.

Although free clinics are one of the most immediate ways to relieve animal suffering, participants highlighted that they address the symptoms rather than the root causes of welfare problems leading to lack of long-term change. *“if you’re not addressing the root causes*, *then you’re not really going to be achieving*, *you know essentially being a band aid kind of project*.*”* P15. This view supports previous research advocating for a move to more prevention focussed programming [3]. Having a free service available to address animal health problems also led to questions over owner motivation to prevent those problems occurring. *“the feeling of some of the staff were that the same horses just kept coming back … they felt that they were very much treating symptoms rather than addressing the causes*.*”* P5. The lack of cost associated with veterinary treatment provided by NGOs had, in some instances, led to owner delays in treatment seeking for serious welfare issues. This potential to cause harm to the animals that were meant to benefit from treatment provision was described by one participant as a significant factor in the organisation’s decision to stop providing a free clinic treatment model. *“with the mobile clinics*, *sometimes*, *because people would know that it would be free*, *people would wait a month to seek treatment because they’d be like*, *well*, *the [NGO] van will be here in a month*, *and then it’ll be free*. *So they had evidence*, *not just that it wasn’t being successful*, *but that potentially*, *there was actually harm*.*”* P27. There were also concerns that an unintended consequence of providing free treatment was a negative impact on local veterinary professionals through loss of business, a concern echoed in other publications [4]. *“I was in a brick kiln in [country] working with one of the private para-vets…and I asked him what his biggest challenges were in his work*. *And he said*, *‘it was when you guys used to turn up and give free treatments*, *and nobody would pay for any of my treatment*.*”* P27. Parallels can also be drawn with the human primary healthcare sector where NGO healthcare provision has been criticised for undermining and fragmenting local health systems [26].

However, in areas where there is no established veterinary infrastructure, NGO clinics may provide the only treatment that animals are able to access. *“just recognising that there are welfare needs*, *and how do you address them right now*, *if you don’t have provision of free care*?*”* P31. Whilst concerns regarding sustainability existed, addressing poor health and injury is the quickest way to alleviate suffering and all participants acknowledged that veterinary treatment was likely to make a large positive impact on the quality of life of the animals receiving treatment. *“who can deny that veterinary clinics are good for the animals that get to see them*!*”* P5. In areas where health problems were severe, it was felt that efforts to address other areas of welfare ahead of or instead of veterinary treatment would achieve limited success. *“saying let’s sit down and talk about aspects of welfare is great but you need to reach a certain level of veterinary support before that tends to happen in my opinion*.*”* P6.

Some organisations used veterinary clinics as an initial approach when entering a new community in order to establish a positive relationship and demonstrate the professionalism and trustworthiness of the organisation. *“providing those services to the communities is*, *is a good introduction method for your ability*. *So I think that’s a very important aspect to include in a community*.*”* P21. This was especially pertinent in areas where equids are not considered a priority species for receiving treatment. *“So free treatments is always used as a gateway to create a win and show them that actually treating the animals improves the body condition*, *improves the life expectancy*, *improves efficiency and work capacity”* P11. Employing a free clinic approach to treat the worst welfare problems in these situations can be used as an avenue to engage with owners about other aspects of welfare and lead on to discussions about preventative care. *“So once we treat and we’ve made the donkey’s life better*, *the livelihood is saved and the community engagement gets even better*. *They get really engaged with us because they know the value ‘these people came here all to help our livelihoods*, *our donkeys so I think we should listen to them about the better harness to prevent wounds*, *I should listen to them about better hoof care’”* P8.

Veterinary clinics can also support other domains such as training and research. Clinics provide an opportunity for vet students to learn skills in recognising and treating common equine health problems and positive handling methods. This was highlighted as being especially important in countries where equines are absent from the veterinary curriculum. Clinics also offer the opportunity for research to be conducted which can form an important part of disease surveillance and prevention. *“the other important thing in veterinary clinics is actually the charities they do support the student research schemes to do surveillance programmes rather than doing it by themselves in most of the cases in collaboration with the government*.*”* P11.

Acknowledging the balance of positive and negative aspects surrounding the provision of veterinary care, some participants suggested that careful and strategic planning and clear communication can minimise some concerns. *“that’s something that you have to be really careful when working with community members and people*, *unfounded expectations*. *And you have to be very clear*, *and therefore you need to have a strategy saying that*, *okay*, *we’re going to be doing this for this amount of time”* P21. Others preferred to use clinics only under certain circumstances or in combination with other approaches. *“I don’t think that long-term that is purely the best way forward*. *In the right time and the right place*.*”* P2. There was also a distinction made between short-term or one-off clinics run by vets flying in from overseas and clinics that build local capacity. *“it would depend how it was provided… if you’re building skills in somebody*, *in a locally trained vet*, *then that is slightly different*. *Obviously*, *then*, *hopefully*, *you’re creating a more sustainable*, *long-term solution and that you’ve got locally skilled people who understand local context and can provide a locally appropriate service*.*”* P32.

#### Para-vets (community animal health workers)

The role of para-vets (sometimes called community animal health workers (CAHWs) or community based welfare advisors) was felt by many participants to be important in contributing to the sustainability of local scale health and welfare provision. *“about 1000 para-vets a year I think were being trained there and they would go back to their communities and they would take all of their knowledge with them”* P4. Accessibility of para-vet services was highlighted as a big advantage to communities. As community-based practitioners, para-vets are available locally and at short notice. *“they are… from within the communities*. *So*, *they can help the communities in need especially in the night hours when all the hospitals and medical schools are closed*.*”* P28. Whereas particularly in rural and remote areas, accessing more formal veterinary services at clinics or health posts can mean a long journey. *“And if a community is lucky*, *he has to travel with the sick animal at times*, *over two kilometres minimum*, *it is very difficult*.*”* P29. It was also mentioned that nomadic communities who are reliant on animal breeding for their livelihood, but often fall outside of the reach of veterinary services, can benefit from para-vet training. The affordability of para-vet services was felt to be an important factor in the success of para-vet programmes. *“if you can make some basic first aids available at the place where they are*, *that is something that could be affordable*.*”* P8. The flexibility of payment that para-vets can offer was preferred by some communities as it was more practically feasible for their livelihoods. *“They can pay anytime*, *the government service provider has to accept the money now*, *you know*, *they don’t have cash economy*. *Farmers can get money when they sell some of their produce during market days*. *They may not have money essentially*. *But the CAHWs can treat them readily and accepts the money next month or next week*.*”* P29. Although limited in scope in comparison to a veterinary professional, para-vets can help reduce the pressure on veterinary services by treating more minor health issues. “*They took the pressure off the team because instead of having to go out*, *keep getting calls from you know ‘my donkey’s got a cut’ that could be treated by the CAHW*. *If the donkey had something more serious they would still go to the CAHW*, *he would assess it and then say ‘yeah I can do that’ or say ‘this is beyond me’ and he would then call the team”* P4. Para-vets can also then act as bridge between communities and veterinarians or other equine service providers to promote preventative healthcare and positive welfare. *“So apart from this*, *we link them with the existing services that we are supporting*, *like vaccination”* P29. In a human health parallel, there has been a rekindling of interest in the use of community health workers to help meet child survival goals, particularly in areas of the world where there is low coverage of primary healthcare professionals [27].

Despite the positive aspects that para-vets can bring, some participants mentioned concerns about regulation of para-vet services, this especially applied to areas where para-vets had greater scope to provide treatments such as administration of drugs by injection. *“some states are giving them three months of training and some states are giving them two years training*, *three years training*. *So it’s very*, *it is a huge variation among different states*.*”* P28. Ethical concerns were also voiced that some individuals profit from a business opportunity and as a consequence the quality of care given can be questionable. *“They end up in under dosing the animals*, *and not giving proper doses…even though it is so crucial*, *it has to be strictly followed that actually they properly operate in particular areas*.*”* P11. Incorrect dosing of animals, leading to potential increases in multidrug resistance, has also been documented in drug retail outlet workers [28]. Regulation of para-vet training, monitoring of service quality and inclusion of veterinary ethics in training were suggested as avenues to ensure that service providers were having the desired positive impact on welfare.

#### General welfare messaging

Although discussed less frequently than some of the other approaches, general welfare messaging had been utilised more frequently recently due to the COVID-19 pandemic impacting NGO’s ability to provide a physical presence in communities. *“So we’ve been trying to send messages to them*, *educational messages…making sure that they understand that even if we’re not there physically*, *that we’re there for them as support*.*”* P24. Amid concerns that welfare may regress during this time, participants described welfare messaging via mobile phones as a way to keep in touch with project participants. *“we send out bulk SMSs*. *So if we need to let them know anything important*, *or we want to give any tips…like with COVID*, *we kept on sending information about how to keep themselves safe”* P23.

It was described that general welfare messages were a good way to reach a large amount of people at a time in a format that was easily accessible and were used in particular to cascade preventative care messages to communities. *“We’re able to reach about 70 to 80% of our target group*, *with this approach*, *we also understand that we can cover also communities that are out of our intervention area*.*”* P30. However one disadvantage was that there was little avenue for following up what the impact of the information was. *“If you had done a radio announcement or broadcast…you had reached like maybe 1000 people but you had no real clear idea of what effect you had on those people”* P4. In the humanitarian sector issues have been raised regarding the utility and function of general messaging such as programme signage, with concerns that increasing individual NGO visibility in order to account to donors may be prioritised over messaging efficacy [29]. Monitoring and evaluation of this type of indirect method is needed to ensure that the desired impact is being created.

#### Microcredit

The formation of microcredit groups was one of the least utilised approaches discussed despite being successfully and widely used across other NGO sectors [30,31]. Some participants expressed their reservations at the prospect of being responsible for others’ finances. *“We’ve done a bit looking at the microcredit but I mean*, *it’s such a minefield for me”* P13. Some felt that such approaches would not be suitable for the cultural context or communities that they worked in. *“we don’t get involved in their financial… it’s too problematic for us to try and have like saving schemes and hold people’s money”* P23.

Success was suggested to be contingent on a cohesive community and was described as more likely to be successful in certain countries in which community savings schemes such as Iddir in Ethiopia are common practice [32]. Those who had undertaken a microcredit approach described the benefits that could be realised when a group of people worked together to invest in improving welfare. Collectively groups could afford items that they never would have been able to purchase alone. *“we had lead people in each little community group we were working with*, *it actually came from their idea… so that was great for trust reasons to let them lead it*. *And when they had enough money then*, *they didn’t wait until everyone had enough*, *just each month when they grouped together they could buy a bit of a harness so they decided who would get the harness that month and they all kept going until everyone got one*.*”* P2.

Despite the potential that microcredit schemes offer, one participant observed a reluctance among donors to offer credit related types of programme. *“A lot of donors are really skittish about that*. *Because*, *you know*, *giving people money – what is that*?! *We are so afraid of giving people money because we think they are going to be*, *that they are going to misuse them”* P21. However, it was pointed out that without access to funding, (such as that provided by seed fund schemes) equine service providers were unlikely to be able to establish themselves as viable, self-sufficient businesses. *“you know*, *people tend to think ‘but we are giving them training*, *that should be enough for them to*, *you know*, *create a large business and work’*! *No… I think*, *the most important aspects for creating businesses are*, *you know*, *the accessibility of funding*.*”* P21.

Equine saving and credit groups, in certain contexts, had far wider societal implications. *“two things which is good*. *One is the equid owners*, *the equid family have a direct access on that front*. *The other thing is that it’s decreased dependency on the local moneylenders”* P25. It was described that before the existence of the NGO saving and credit group, borrowing of money for emergency expenses was only available from high interest rate local moneylenders. When the equid owners were unable to pay, they entered a cycle of bonded labour; the role of moneylending in the persistence of the poverty cycle has also been highlighted in other NGO sectors [33]. The avoidance of bonded labour was mentioned as the primary reason to consider starting a microcredit scheme by an NGO that worked mainly with owners in the brick kiln industry. *“So the brick kiln owner exploits the people who want money for emergencies*, *it might be a child’s medical emergency or a marriage or a death in the family… the small little group can help them in crisis*. *That is something we should also try I think*. *I have heard about and there’s a possibility we should bring it into the programme*, *I feel strongly”* P8.

#### Education

Across organisations, a common theme in successful educational approaches was interactive, practical learning. Whether it was in the context of training vet students, government officials, owners at veterinary clinics or staff at behavioural workshops, it was felt that if learners could carry out practical activities themselves, the educational message would be better remembered and overall learning would be more effective. *“personally I would always go for practical training*, *I don’t like chalk and talk in a classroom*, *it is all about getting out and seeing it on the ground”* P3. It has been documented that practical demonstrations of novel management techniques can accomplish recognition of the technique’s benefits within the owner population [34]. Hands on contact and demonstrations with animals were seen as being particularly memorable. *“the one that was most successful*, *I think was where we were able to do a practical session and go out and actually handle working equids and show them*.*”* P14. This also applied to training in new techniques for members of NGO staff. *“there have been animals I guess that have changed the way a whole team might think*.*”* P6.

Practical participation when introducing owners to basic management practices such as wound cleaning can be especially important as individuals need to be able to repeat the techniques unsupervised outside of a clinical setting. *“And when you do the veterinary care*, *okay*, *that’s when I started introducing the method of*, *I do it for you halfway*, *and I tell you what to do*, *and I sit there and watch you*, *and you do it yourself*.*”* P22. Practical participation was also mentioned as being useful as low levels of literacy in some equid owning communities mean that other commonly used methods of communication such as written welfare messaging are unsuitable. One participant also described a higher level of community engagement for practical tasks. *“not just words*, *because they’re very much you know*, *tired*, *bored by the extension messages coming from government and talks*.*”* P29. Interactive exercises with owners were also discussed as a method of encouraging owners to be able to visualise things from the perspective of their equid. *“we prepare a sack full of sand… and then make to carry the owners of the donkey and then feel how heavy*. *See look*. *This is what you’re actually loading your donkey*. *Can you feel how heavy it is*?*”* P11. Empathy has been directly implicated as an influence on animal welfare [35] and studies have demonstrated relationships between higher levels of empathy towards working horses and more accurate perception of their pain [13]. It has been suggested that in decisions relating to welfare, owner empathy may play a bigger role than socio-economic status [36] therefore the fostering of empathy in owners has been recommended as a method for the improvement of equid welfare [13].

The development of empathy was also a large focus of school directed education programmes. NGOs varied in their level of school education offered; one charity had a dedicated education team specifically to develop humane education programmes delivered across their projects. Other NGOs described running after school welfare clubs where fun activities such as singing, art and drama conveyed equine welfare messages. It was felt that children who were aware of animal welfare needs would grow up to be more empathetic owners, taking better care of their animals. *“One key approach that I think should be adopted is focusing on child education*, *you know*, *from the early years on animal welfare*, *because these are the future generation*.*”* P26. At a young age it was felt that children were better able to take on board new perspectives than adults who may be more entrenched in their beliefs. *“it is fundamental*, *working with children… you know*, *because children really*, *really can change their mindset*.*”* P21.

It was described that the reach of children’s education programmes extended beyond just the children themselves, an effect also seen in the field of child-centred environmental education [37]. The information given was disseminated by children to their parents and friends and generated awareness of future NGO events and initiatives. *“not only will they then grow up to be members of the community*, *but they’re also going home and talking about animal welfare to their parents*. *And so also potentially talking about [our organisation] if [we are] coming or… other organisations that have been coming into the communities so that we get better uptake of community training initiatives that we run*.*”* P31. Many of the children involved in schools programmes already have roles in taking care of their family’s equid and so the information given can be of direct benefit. *“their neighbours*, *their families own an animal*, *and then they can influence back their parents to*, *to at least provide the necessary needs for animals*. *And these kids*, *you know*, *they provide some water to the animals*, *they groom them*.*”* P29. One difficulty of school education was being able to measure the impact that programmes are having. *“we have quite a large school education programme… it would be really great to be able to demonstrate the benefits that aren’t just anecdotal or theoretical*. *But yeah again*, *that’s a really difficult thing”* P31. The lack of direct evidence from monitoring and evaluation of school education initiatives was, for some, a discouraging factor for investing in child focussed programming. *“I think there are some things that take absolutely ages to be of benefit like the education of kids in schools and stuff*, *that sounds like something that is really really useful to do but in terms of success…you’ve got to be watching that project for what 15 odd years before you get an idea really of how successful it is*.*”* P2.

#### Community participatory exercises

A focus on the human drivers of welfare had seen an increase in the use of community participatory exercises such as participatory rural appraisal (PRA) by working equid NGOs. PRA uses a variety of participatory tools such as transect walks, scoring and ranking exercises, informal mapping and the creation of diagrams to enable local people to share their perspectives and knowledge [38]. PRA approaches are well established in epidemiological research, international development and environmental education and are now becoming more frequently used in animal welfare initiatives [39–42]. Exercises are often pictorially based in order to ensure that group members with low levels of literacy are not excluded from participating. Participants described participatory approaches as an effective way of engaging individuals from the very beginning of the initiative process. Exercises were especially useful for identifying community views on priority welfare issues. Upjohn et al. [34] highlighted that priority welfare issues for people in target communities are not necessarily the same as those identified by an NGO, and this was echoed by participants. *“actually the owners are not prioritising the kinds of issues that are implicitly assumed to be priority issues within those interventions”* P32. Understanding these differences in perception can avoid previously seen scenarios where a mismatch in priorities has led to the failure of an implemented initiative through lack of community engagement. *“when we started training*, *we see the impact is very limited*, *because we didn’t involve the communities”* P16.

It was described that approaches such as PRA have enabled a move away from previously used techniques where information was imparted to the target community (with purely an educational focus) but local people did not participate in the knowledge generation, share their insights or have any ownership over the learning process and outcomes generated [38]. Higher levels of engagement and investment in initiatives was one of the biggest advantages discussed regarding the use of participatory approaches. *“they feel included and more invested”* P21. Participatory exercises are designed to allow individuals to share their local knowledge, perspectives and opinions. *“you manage to have them on board so you are not the teacher teaching them*, *but having them collaborating with you”* P1. Utilising these opinions to come to a shared agreement regarding the direction of welfare focussed efforts enhanced investment in the subsequent initiative. *“if someone comes up with an idea they always try harder to make it work because it is their idea and they take it forward more*.*”* P2. Enabling communities to develop their own strategies was felt to empower communities and ensure that future actions were appropriate and tailored to the local context. *“instead of us telling them what to do*, *we try to get them to facilitate each other in terms of recognising what are the issues with animal welfare and what they can do about it*. *So we are empowering them and the answers came from them*.*”* P19. Another advantage of participatory exercises discussed was their ability to identify the good practices that communities already have established. Highlighting these practices not only reinforced positive welfare aspects within sessions but allowed these practices to be built on to facilitate further welfare improvement. “*in that workshop*, *they got the communities to identify what they already did that was really good*. *And that they wanted to kind of keep…*. *And then they got the communities to identify what they then wanted to change*” P27.

The long-term impact of initiatives born though participatory techniques was also felt by participants to be better. *“it creates greater and more sustainable behaviour change when you do it in partnership with people*.*”* P7. This was also linked to the ability to use participatory exercises to identify and discuss the root causes of welfare problems. *“I think that that’s very important to find out*, *you know*, *what are the issues and not just the symptoms but actually delving down into what are the causes of something”* P15. Being able to then address the root causes of welfare problems represented an avenue for sustainable welfare improvement.

#### Skills initiatives

These initiatives aim to train individuals in specialist (non-veterinary) skills such as farriery, saddlery and harnessing in order to create businesses providing those services to the community. Participants described: *“education and skills initiative…this is a very crucial action in creating awareness and equipping the communities…with basic knowledge and skills”* P11. Studies have reported as many as 78% of equids in a population affected by working equipment related injuries, and these skills initiatives can be especially useful in such areas [43]. *“they taught the para-vets how to make basic harness*, *just pack saddles and very basic harness but that’s had a massive effect*. *The knock-on effect from that was tremendous”* P4. With huge variability seen in the standards of local service providers [14], creating a network of highly trained providers can ensure that both animals and owners are receiving high quality services, helping to maintain welfare within the community. *“where we were based…pretty much all of the owners would use one of the farriers that we trained*. *I think that was so successful”* P2.

Most initiatives aim to train members of a community so that they can become full-time service providers within their community. *“you want for them to work with you to be viable local businesses*.*”* P21. However for this to happen, individuals need to have a financially feasible business which some participants described as difficult to establish. *“People were really keen to learn how to make stuff but trying to then sell it was quite hard because it was obviously way more expensive than like old clothing or the rags people were using”* P2. In order for initiatives to be successful, there needed to be a demand within the community for the service that was being offered. *“just because you create a service doesn’t necessarily mean that you fixed the problem”* P32. It was felt that some previously run initiatives had been unsuccessful because the skills imparted by NGOs, and services subsequently offered, were not targeting priority issues for local people and resulted in a lack of investment. Differences in welfare priorities between communities and NGOs have been previously noted [34]. Due to this need for service demand, some organisations only focussed on upskilling existing service providers within communities. *“we don’t want to start from zero we just want to start where there are already service providers*.*”* P26. In this scenario individuals are already established which removes the barrier of creating both service demand and local infrastructure. *“there was already this setup to use that service so it wasn’t like this big stepping stone it was just improving a service that was already there*.*”* P2. Relatively small changes to the operations of these individuals can then have widespread effects within a community.

Another challenge discussed was dropout of individuals enrolled in training courses. *“you’re taking 10 people… at the end of the year you are left with six people*. *So my concern is those four people why are they leaving*?*”* P22. As courses are intensive to teach, a substantial amount of resources and time are invested in candidates and participant dropout was a significant loss. Trained individuals subsequently leaving their communities was another area of concern discussed which was felt to hinder the success of initiatives looking to build networks of local service providers within an area. Participants highlighted the need for continued support for skills initiatives, particularly for ensuring the longevity of service provision. Previously used models for the delivery of skills initiatives saw UK based professionals teaching courses for short time periods in target countries. This was not felt to be adequate for the long-term maintenance of these skills within communities. *“If you do it in a way that’s shipping them in and then inject some knowledge and go away again*, *I have found that those are very unsuccessful although they have a lot of excitement at the time*.*”* P5. Many NGOs have now shifted to in-country delivery of skills training programmes which allows courses to be taught by local professionals, tailored to the local context and provides increased scope for ongoing support.

#### Barriers to welfare improvement

Although participants discussed the strengths and weaknesses of the different initiatives used, similarities emerged in factors that impacted NGO programming regardless of the approach taken. These inherent challenges when trying to engage in welfare improvement initiatives cross-cut initiative categories and were widely described by participants working across countries.

#### Equid status

One such barrier, described across many institutions, was people’s attitude towards equids, both at the community and policymaking level. This was especially apparent in the case of donkeys who, across countries, are frequently associated with low status. “*even now in [this country] donkeys are not considered great… most of the people*, *not even the veterinarians want to go and treat the donkey*, *thinking it is a very neglected abused animal*.*”* P8. It was felt that donkeys’ association with menial or household work and comparatively lower sale price influenced opinion of their ‘worth’; this has also been documented in other research studies [12,44]. *“Donkeys are generally not taken care of well by the communities because they are cheaper and they are let loose for grazing”* P28. This results in a systematic lack of basic care for donkeys, which is not reflected for other equine species. The perception within communities that donkeys are low maintenance and ‘take care of themselves’ was frequently mentioned. *“malnutrition is one of the problems because as I said*, *donkeys their priority is so down some of them don’t even feed them*. *Because donkeys normally… get whatever they get from roaming that is how they live”* P11.

Due to donkeys’ low status, encouraging consideration of their welfare needs can be met with incredulity and sometimes derision. *“apparently lots of them turned up just because they couldn’t believe there was going to be a project about donkeys and they just came to find out*, *because that was the ultimate craziest thing [a project about] being nice to a donkey*.*”* P5. The association of equids with low status can also extend to professions related to equids such as farriery. This perception and a lack of respect associated with the profession can prevent the recruitment and retention of individuals to farriery training programmes. *“it’s not a very respected profession*. *So there’s not much pride in being a farrier*. *Often*, *the animal’s feet are described as dirty*, *farriery is therefore described as a dirty job”* P27. The cultural status of equids in some areas also affected their treatment by vets. *“so getting them to engage with the idea of equids as a species you want to be a vet for is often challenging*.*”* P32. The potential reasons for this were discussed and included: a lack of equid inclusion in veterinary curricula, wider societal views that do not prioritise equids and a lack of uptake of services as some owners are reluctant to pay for treatment for equids. Despite their importance to livelihoods and domestic tasks, the value of equids and the activities they perform are not always recognised by owners. *“I would definitely hear owners saying*, *there’s no point*, *you know*, *there’s no point putting money into donkeys because they die anyway*.*”* P32. Other livestock (those that provide a more direct benefit such as milk or meat) are often considered to be more important and as a consequence receive more care and veterinary attention in situations where financial resources are scarce [45].

This situation is also mirrored in the advocacy struggles encountered by NGO staff when trying to influence policy changes in favour of working equids. *“the invisible equine within the development agenda is a problem and then animal welfare is also a bit of a blind spot*.*”* P15. A lack of recognition of the value of equids by governments and policymakers both at local and national levels was described. *“And when you actually look at the population statistics with FAO*, *some countries don’t even register that they’ve actually got working equids*.*”* P14. It was felt that research demonstrating the contribution that equids make could favourably influence their status both in the eyes of the public and policymakers. *“we need to bring a change in the attitude of the general public of how good and innocent and hard-working an animal it is*. *How is their contribution to the economy”* P8. There was understanding that other important societal issues will compete for government funds and legislative action but issues around working equids appeared to be low on policymakers’ list of priorities. *“In Africa you’ve got poverty*, *unemployment*, *HIV/AIDS*. *Huge*, *these are huge cross-cutting issues*. *They equally demand funding and skills”* P15. The indirect benefits that working equids provide make it difficult to quantify their value to a country’s economy [4]; and in countries where working equid populations are decreasing (as mechanisation becomes more widespread), there were concerns that their position is viewed as irrelevant to a countries’ future growth. *“the population of equids as per the government census has decreased significantly*… *so the government is not focusing on it…the equids are not a priority”* P28.

Working equids’ exclusion from livestock policies was a barrier that was discussed across organisations and NGOs, and advocating for their inclusion was a focus of their programming. Successful advocacy resulted in the acknowledgement of equids as ‘working livestock’ contributing to food security by the UN Committee of Food Security in 2016 [46]. Recognising working equids as livestock covered within national livestock policy definitions can have a range of beneficial effects [47]. These include access to national animal health strategies and services such as vaccination campaigns, disease surveillance and emergency relief schemes [45]. One participant highlighted the need for equid inclusion in current livestock disease control strategies, particularly in the case of zoonotic diseases that pose a serious risk to human health such as anthrax. *“it affects cattle*, *it affects sheep*, *goats*, *horse*, *donkey*, *human*, *and other animals too*. *The funny thing is the government actually do the vaccine against cattle*, *but they don’t vaccinate against donkeys… they’re actually opening a pocket*, *the problem is still there”* P11. Leaving equids unvaccinated can create reservoirs of disease in populations that can then aid transmission to other animals or potentially humans.

#### Access

Accessibility was raised as a barrier to the success of welfare initiatives, both regarding access to communities and access to all individuals responsible for maintaining equid welfare. It was discussed that some equid owning communities live in very remote rural areas where physical access is limited by the environment, such as mountain communities. By virtue of their position, these are often the communities that rely heavily on working equids for many roles including the transport of people and goods across difficult to navigate terrain where the use of a vehicle is not possible [48]. But nevertheless, for NGOs attempting to reach these communities, access is a challenge. *“I think it’s more their location makes it quite difficult for even our teams that are even based near them to even be able to access them*, *to bring the stuff that they need to kind of do the work*. *And it’s usually the worst welfare conditions are in the areas that are the most remote*.*”* P17. The COVID-19 pandemic has further exacerbated issues surrounding community access, a finding that has also been documented across NGO sectors [49]. It was described that remote communities also have problems accessing veterinary services due to their distance. This was especially mentioned in the case of brick kilns which, due to pollution regulations, are situated away from urban areas. *“even if they want to call veterinary services*, *people don’t come because of the distance and the remoteness of the place*.*”* P8.

Access to the full spectrum of individuals involved in the day-to-day care of equids was also another challenge highlighted by professionals. This is especially pertinent to cultures where the gender division of labour can mean that the individual working with the animal is not necessarily the person who undertakes routine equid management or attends an animal clinic. “*So for example in [country] you often have children bring the donkey to the clinic for treatment and I remember on a particular occasion a young girl had brought a donkey*, *it had abscesses in both front feet and the vet said ‘I thought I told you*, *you weren’t to work the donkey’*, *clearly by the foot pads the donkey had been worked in between the treatments and the girl’s reply through translation was ‘I know what you said but how do I tell my father*?*’”* P6. Many participants described the need for programmatic activities such as meetings, workshops and educational events to include all individuals responsible for equid care. However, this was sometimes challenging; cultural norms in some areas can prohibit mixed-gender community participation and different time constraints across genders can mean that certain individuals are not available to take part in NGO programmes. In these situations NGOs can struggle to include the views of, or impart knowledge to all of the people necessary to make improving welfare successful.

#### A lack of equid trained vets

A frequently highlighted barrier to the success of equid welfare projects was the absence of training related to equines in the curriculum of veterinarians across countries. This has resulted in local veterinary service providers who do not have the specialist knowledge or skills to treat equine disease or injury. As many communities do not have the option to seek treatment for their animals elsewhere, this lack of access to appropriate treatment further compounds the poor welfare state of working equids. *“If they manage to get somebody there for the veterinary services*, *the person who deals with the case aren’t trained or have the skills to deal with donkeys and mules*.*”* P8. While production animals appear to be the focus of much of the veterinary curriculum, equids in some areas are not commonly encountered by vets. *“5th year vet students and they’ve not touched a horse*, *they’re scared of it*!*”* P13. In some areas, even if there is provision made for the veterinary treatment of horses, knowledge of donkey treatment is severely lacking. It is common that no differentiation is made between treatments for different equid species. *“Another problem is that the majority of people think that donkeys are just a small horse and they have the same needs”* P1.

Across NGOs there was a programming focus on working in partnership with veterinary universities to bring elements of equine training to the curriculum. *“So we’ve tried to do a 101 working equid curriculum*. *That might be ideally*, *teachers that will take this on board and make them their own and listen to our recordings*. *And we’re building slowly*, *sort of a database that they can have*.*”* P31. Programmes where vet students can gain experience in the handling and treatment of equids were also run by NGOs with the hope of ensuring a better coverage of equid trained vets returning to their regions. *“when the vets come and work with us*, *they can come with us in the field as part of the internship programme*, *start touching the donkeys and injecting donkeys*, *in our presence so it builds their confidence*.*”* P8. Equid trained para-vets can also help to fill this gap in provision.

#### Monitoring and evaluation challenges

Many participants described an increased focus on monitoring and evaluation (M&E) of initiatives. This focus parallels changes seen far earlier in the field of international development where the need for accountability in the use of governmental and institutional funding resulted in more rigorous reporting and evaluation systems [50]. M&E was not perceived negatively by participants, all participants were in favour of tracking the progress of initiatives and felt that M&E was important to know how much of an impact their projects were making. However comprehensive and structured M&E processes were described across virtually all organisations as a relatively new introduction and rigorous M&E across the different approaches now utilised by organisations was highlighted as a challenge. *“I would say this is relatively new to the programme so…it is a little bit trial and error*. *And it’s also quite new to our teams*. *So there’s a lot of work in that”* P13. Apprehension was expressed that the increased need for formal evaluation has put extra pressure and workload onto those collecting M&E data at ground level. *“So there’s a lot of paperwork which the field team hates*!*”* P23. It also carried the risk that the time spent evaluating projects would result in less time devoted to carrying out the activities that would create desired change, a focus on upwards accountability seen in other NGO sectors [29]. *“suddenly we went from actually going out there helping donkeys to spending all our time trying to prove we were helping donkeys”* P4. With the implementation of new frameworks came concern that the expectations were not compatible with the practicalities of situations on the ground. *“it was a very sort of tricky conversation to say*, *you know*, *we are not sure if this is realistic”* P32.

Participants spoke about the need to use indicators that are ‘meaningful’. Historically numbers of animals reached by NGO programmes were the most common indicator of success measured [5]. However, this did not capture the impact that initiatives had on those animals reached and whether this led to any type of long-term welfare benefit. *“Instead of just saying we treated*, *I think it used to be like 360*,*000 donkeys a year at that time*, *as [a colleague] quite rightly said ‘that could be one donkey 360*,*000 times because you treated it really badly and haven’t done anything for it*!*’ What does it mean*? *It doesn’t mean anything”* P4. It was also raised that what is deemed a meaningful indicator may differ by area. *“So we also have the balance between indicators that Western developed countries think of as indicators versus what are really useful indicators here in more developing countries*.*”* P19.

With the focus shifting from numbers of animals reached to longer-term success, interviewees expressed difficulties in finding the most effective indicators to measure complex outcomes that can be influenced by multiple factors. This was especially true of multidisciplinary initiatives which seek to improve equid welfare indirectly through livelihood enhancement. *“I think the biggest challenge with that is that it is successful*, *but it’s been hard to measure how… how can you then know that that’s directly improving equine welfare*, *if you’re improving someone’s livelihood”* P20. It was also a concern that external factors could lead to an unrepresentative negative evaluation. *“actually measuring real impact*, *particularly when…in the middle of your three years*, *there’s a massive drought and people lose their livestock and body condition goes right down*. *You’ve been doing these beautiful welfare assessments and then suddenly… there’s so much else that how do you then have all this data and have the thousand caveats that come with it*?*”* P13.

However one participant suggested that the perceived difficulties surrounding comprehensive M&E can be unfounded. “*one of the myths is that M&E is expensive and complicated and it certainly doesn’t have to be*. *You can do it at a lot of different levels and I think that often the baby is thrown out with the bath water because people think you have to do so much when really you can still gain a lot of information from a smaller but dedicated and strategic approach*.” P5. It was suggested that smaller changes, more reflective of the incremental nature of changes made by welfare initiatives could be a more suitable avenue than trying to immediately link initiatives to direct welfare improvement that may take far longer to be realised. *“I think there is not enough emphasis placed on smaller indicators and looking at success in other ways…*. *So for example provision of water could be a small one*, *you could say 100% of people weren’t providing water all the time that their animals were stabled and now 80% are*, *and although that might not lead all the way through to animal-based indicators of improvements in dehydration say*, *you’ve got those human behaviour indicators of what they’re doing”* P5.

## Conclusion

The results highlight some of the major advantages and limitations of the variety of approaches used by equid welfare NGOs. The study provides an informed insight into potential factors to consider when designing future welfare initiatives. Discussion with experienced NGO staff from multiple roles has allowed a wide range of perspectives to be documented and has revealed the division of opinions regarding some of the approaches utilised by organisations. It has also demonstrated some of the current trends in the sector and highlighted approaches that have worked particularly well in certain contexts. Striking a balance appears to be a strong theme, at many levels. Balancing top-down versus bottom-up approaches was a frequently voiced concern, as was balancing the utility of certain approaches against associated factors warranting caution. Combinations of approaches that were felt be complementary provided a balance that drew on the strengths and mitigated for the weaknesses of different approaches. The need to tailor the approaches used to individual contexts was also raised and is especially relevant to INGOs working across multiple countries, cultures and political structures as some approaches were deemed unsuitable or unlikely to work in local environments. The collaborative pooling of experience across different NGOs could aid future programme planning, help make welfare initiatives more effective and provide a framework for NGOs in other fields to learn from each other’s experiences and collective knowledge.

## Supporting information

S1 File Semi structured interview themes. Participant document setting out the themes of the interview.
(DOCX)

S2 File Approaches to improving working equid welfare. Participant document setting out the most commonly used programmatic approaches.
(DOCX)
