# Supplementary material for: “What can we do to actually reach all these animals?” Evaluating approaches to improving working equid welfare
Source: PLoS One. 2022 Sep 9;17(9):e0273972. doi: 10.1371/journal.pone.0273972 (PMC9462723; doi:10.1371/journal.pone.0273972)
Supplement: S2 File — Participant document setting out the most commonly used programmatic approaches. (DOCX) [file pone.0273972.s002.docx]

**Approaches to improving working equid welfare**:

This document lists a number of approaches to improving working equid welfare that you might be familiar with. We would like to give you the time to consider these approaches before the interview. During the interview we will ask your opinions on the approaches listed and what you feel might be advantages or disadvantages of these methods.

- **Veterinary clinics**

The provision of free (or subsidised) veterinary services ranging from preventative care, vaccination and disease surveillance programmes to emergency treatment.

- **Educational and skills initiatives**

These include introducing skills into communities through training individuals in areas such as farriery and saddlery, running sessions on specific topics such as wound care or handling methods, educational programs aimed at children of school age.

- **Training CAHWs (Community Animal Health Workers) or para-vets to work in communities**

The training of individuals in aspects of animal health to remain in communities that do not have veterinary coverage, enabling them to treat common equid health problems.

- **Community Participatory Exercises**

Exercises that involve the owners of working equids in the analysis of equid welfare issues. This could take the form of transect walks, scoring and ranking exercises or drawing diagrams to enable owners to share their perspectives and knowledge.

- **General welfare messages**

Messages promoting positive equid welfare and the associated benefits that having a healthy equid can bring. These could be delivered at community meetings, via radio, handouts or signage.

- **Entertainment with a welfare message**

Performances for entertainment such as songs or drama that contain a message emphasising positive equid welfare.

- **Microcredit programs and savings associations**

Collective savings schemes that enable participating owners to borrow money from the collective fund for purchases such as repairing equipment or buying a new animal.

- **Policy changes at local, national or international level**

Advocacy, lobbying and influencing future policy regarding working equids such as protective laws and legislation.

- **Research**

Equid focussed research projects aiming to fill knowledge gaps in the information known about welfare. The results of research projects may be used to plan future welfare initiatives.

- **Option for other approaches to be identified by interviewee**

If you can think of any other approaches to improving working equid welfare please bring these up during the interview.
